# Transcriptional profiling of murine macrophages stimulated with cartilage fragments revealed a strategy for treatment of progressive osteoarthritis

**DOI:** 10.1038/s41598-020-64515-1

**Published:** 2020-05-05

**Authors:** Masanari Hamasaki, Mohamad Alaa Terkawi, Tomohiro Onodera, Yuan Tian, Taku Ebata, Gen Matsumae, Hend Alhasan, Daisuke Takahashi, Norimasa Iwasaki

**Affiliations:** 1 Department of Orthopedic Surgery, Faculty of Medicine and Graduate School of Medicine, Hokkaido University, Kita-15, Nish-7, Kita-ku, Sapporo, 060-8638 Japan; 2 Global Institution for Collaborative Research and Education (GI-CoRE), Frontier Research Center for Advanced Material and Life Science Bldg No 2, Hokkaido University, Sapporo, Japan

**Keywords:** Osteoarthritis, Osteoarthritis

## Abstract

Accumulating evidence suggests that synovitis is associated with osteoarthritic process. Macrophages play principal role in development of synovitis. Our earlier study suggests that interaction between cartilage fragments and macrophages exacerbates osteoarthritic process. However, molecular mechanisms by which cartilage fragments trigger cellular responses remain to be investigated. Therefore, the current study aims at analyzing molecular response of macrophages to cartilage fragments. To this end, we analyzed the transcriptional profiling of murine macrophages exposed to cartilage fragments by RNA sequencing. A total 153 genes were differentially upregulated, and 105 genes were down-regulated in response to cartilage fragments. Bioinformatic analysis revealed that the most significantly enriched terms of the upregulated genes included scavenger receptor activity, integrin binding activity, TNF signaling, and toll-like receptor signaling. To further confirm our results, immunohistochemical staining was performed to detected regulated molecules in synovial tissues of OA patients. In consistence with RNA-seq results, MARCO, TLR2 and ITGα5 were mainly detected in the intima lining layer of synovial tissues. Moreover, blockade of TLR2 or ITGα5 but not Marco using specific antibody significantly reduced production of TNF-α in stimulated macrophages by cartilage fragments. Our data suggested that blocking TLR2 or ITGα5 might be promising therapeutic strategy for treating progressive osteoarthritis.

## Introduction

Osteoarthritis (OA) is a progressive joint disease that causes chronic pain and physical disability in elderly people. The disease is highly prevalent with worldwide incidence exceeding 250 million people^1^. With increasingly aging population, the disease is expected to be more prevalent than in the previous decades. To date, there is no effective disease-modifying therapy and arthroplasty is the only choice to relief symptom and restore the function of joints. Discovery of effective pharmacologic agents for treatment of OA is required a better understanding of the molecular pathways and signals responsible for OA progression^2^.

OA is traditionally characterized by articular cartilage degradation, subchondral bone remodeling, synovial inflammation (synovitis), and osteophyte formation^3^. The pathogenesis is complex and includes mechanical, inflammatory, and metabolic factors, which ultimately lead to structural alteration of the synovial joint. Macrophage accumulation in the intimal lining is the primary morphological features of synovitis. It is evident that infiltrated macrophages play a significant role in the progression of OA through initiating phagocytosis, forming multinucleated giant cells and producing cytokines and other inflammatory mediators. Activated macrophages alter the function of synovial fibroblasts and chondrocytes resulting in an increase in inflammation and production of catabolic factors in the joint^4,5^.

Tumor necrosis factor (TNF-α) is one of factors derived from macrophage that can promote catabolism in joint via suppression of the synthesis of proteoglycan and type II collagen in chondrocytes and enhancing the production of proinflammatory and procatabolic mediators^6^. Increasing evidence suggests that gut microbiota-derived endotoxins are involved in OA pathogenesis through activating joint tissue macrophages resulting in chronic inflammation in joint^7^. On the other hand, cartilage fragments and molecules from degraded hyaline cartilage are thought to contribute to in the development of synovial inflammation through activating macrophage inflammasome pathways^8,9^. In fact, components from damaged extracellular matrix act as danger-associated molecular patterns (DAMPs) that activate synovial macrophages to produce inflammatory mediators^10^. Likewise, an earlier studies showed that intraarticular injection of cartilage fragments induces synovitis and osteoarthritic changes in animal models^11^. Therefore, it becomes pertinent to examine the possible role of cartilage fragments in the etiology and progression of osteoarthritic process. Our earlier study showed that macrophages cultured with cartilage fragments produce inflammatory mediators that modulate the function of chondrocytes^12^. However, the molecular mechanisms by which cartilage fragments trigger macrophage responses remain to be investigated. Therefore, the current study aims at understanding the molecular response of macrophages to cartilage fragments as step towards identifying molecular candidates for potential therapies of OA. We analyzed the transcriptional profiling of mice macrophages stimulated by cartilage fragments using an RNA-seq approach. Our data provide a new insight into the molecular pathogenesis of osteoarthritis and shed light on new molecular candidates for therapeutic intervention.

## Results

### Transcriptional profiling of macrophages stimulated by cartilage fragments

**Figure 1:**
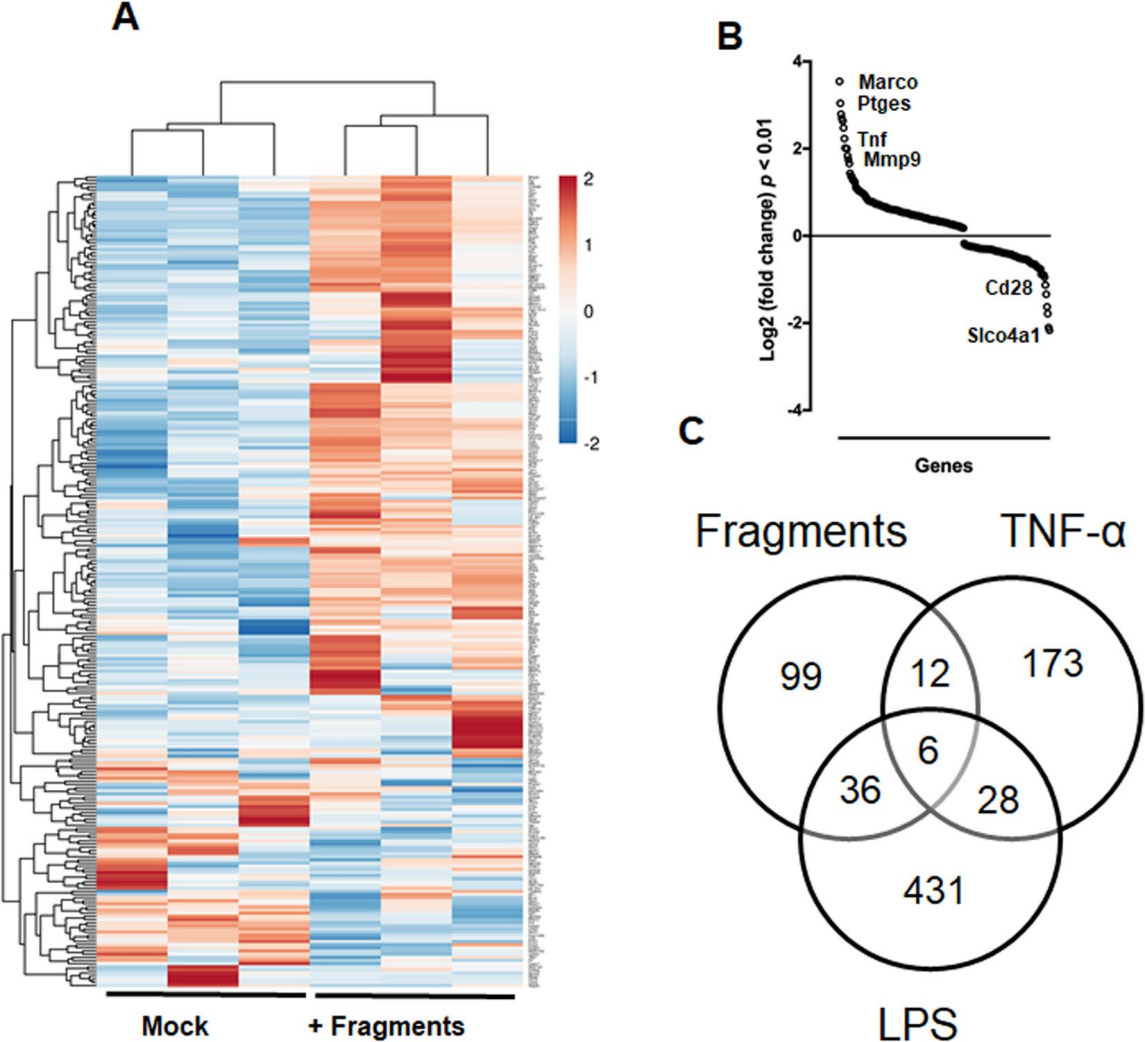
Gene expression profile of macrophages stimulated by cartilage fragments. (**A**) Scatter plot analysis for transcript expression levels of significantly up-or down-regulated genes in macrophages response to cartilage fragments (p < 0.05) (n = 3). (**B**) Hierarchical clustering for the expression of significantly up-and-down-regulated genes in macrophages (p < 0.05). A scale bar for intense color change from −2 or below indicated by blue color or 2 or above indicated by red. (**C**) Venn diagram analysis for the numbers of genes which are significantly upregulated in response to cartilage fragments, LPS and TNF-α.

Bone marrow derived-macrophages (BMMs) were cultured with cartilage fragments for 24 hours and their transcriptional profiles were analyzed by RNA-seq approach. Differentially expressed genes were determined by comparing gene expression of macrophages cultured with cartilage fragments to those of control macrophages. Of note, 153 genes were differentially upregulated and 105 genes were downregulated in response to cartilage fragments (Fig. 1A,B and Table S1). RNA-Seq data were next validated by performing qRT-PCR for upregulated genes, including Tnf, Icam1, Mmp14, Tlr2, Itga5, and Irak3. Of note, the gene expressions of these genes were significantly elevated in macrophages cultured with cartilage fragments as compared to control macrophages (Fig. S2A). In line with these results, the gene expressions of these genes were significantly increased in peritoneal macrophages cultured with cartilage fragments (Fig. S2B). These results suggested that BMMs and tissues resident macrophages may exhibit similar molecular responses to cartilage fragments. We further evaluated the activation state of macrophages by comparing the transcriptional profile of activated macrophages by cartilage fragments to that activated by LPS or recombinant TNF-α^13,14^. A Venn diagram showed that 42 and 18 shared genes were present in the activated macrophages by cartilage fragments and LPS activation, and recombinant TNF-α activation states, respectively (Fig. 1C). These results revealed that macrophages expressed a unique gene set in response to cartilage fragments, which may reflect the pathogenesis of synovitis in OA.

### Functional and biological characterizations of macrophage response to cartilage fragments

**Figure 2:**
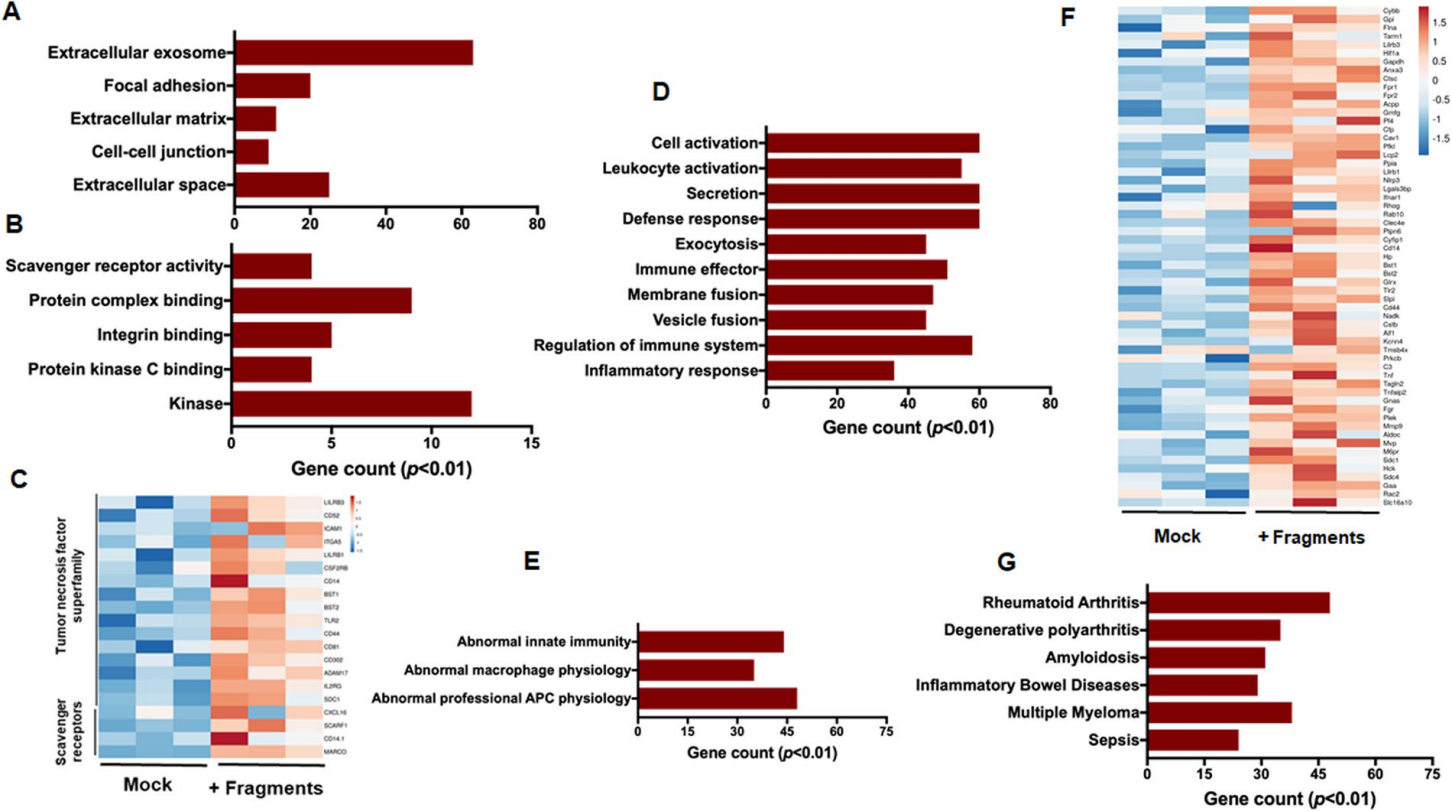
Gene enrichment analysis of differentially expressed genes in macrophages stimulated by cartilage fragments. (**A**) GO terms for cellular component. (**B**) GO terms for molecular function. (**C**) Heat map for clustered gene family. (**D**) GO terms for biological process. (**E**) GO terms for mouse response phenotypes. (**F**) Heat map for the transcript expression levels of enriched genes in inflammation. A scale bar for intense color change from −1.5 or below indicated by blue color or 1.5 or above indicated by red. (**G**) GO terms for human diseases.

GO enrichment analysis for differentially expressed genes was performed to draw an image for the regulatory networks and functional relevance of this biological response. GO enrichment analysis revealed that up-regulated genes were mainly associated with immune process, including extracellular exosome for cellular components, and scavenger receptor activity and integrin binding for molecular function terms, and tumor necrosis factor superfamily for gene family terms (Fig. 2A–C and Table S3). Likewise, the most significantly enriched terms were leukocyte activation for biological process and abnormal innate immunity for mouse phenotypes (Fig. 2D,E and Table S4). It is noteworthy that the macrophage stimulated with cartilage fragments expressed number of genes that are involved in the chronic inflammatory processes including allograft inflammatory factor 1 (Aif1), Tnf and NLR family pyrin domain containing 3 (Nlrp3) as well as hypoxia-inducible factor 1α (Hif-1α) (Fig. 2F). The activation of NLRP3 by cellular stress leads to activation of the inflammasome which is associated with a number of autoinflammatory syndromes and autoimmune diseases. In consistence with these data, the upregulated genes were most significantly enriched in rheumatoid arthritis, degenerative polyarthritis and inflammatory bowel diseases (Fig. 2G and Table S5). On the other hand, down-regulated genes were mainly enriched in cholesterol binding, antigen processing and presentation of exogenous peptide antigen via MHC class II, positive regulation of interferon-beta biosynthetic process, and cholesterol efflux for molecular function and biological process terms (data not shown). Taken together, our results suggest that exposure of macrophages to cartilage fragments leads to production of distinct arrays of inflammatory modulators associated with development of synovitis.

### Pathways and transcriptional factors of macrophages stimulated by cartilage fragments

**Figure 3:**
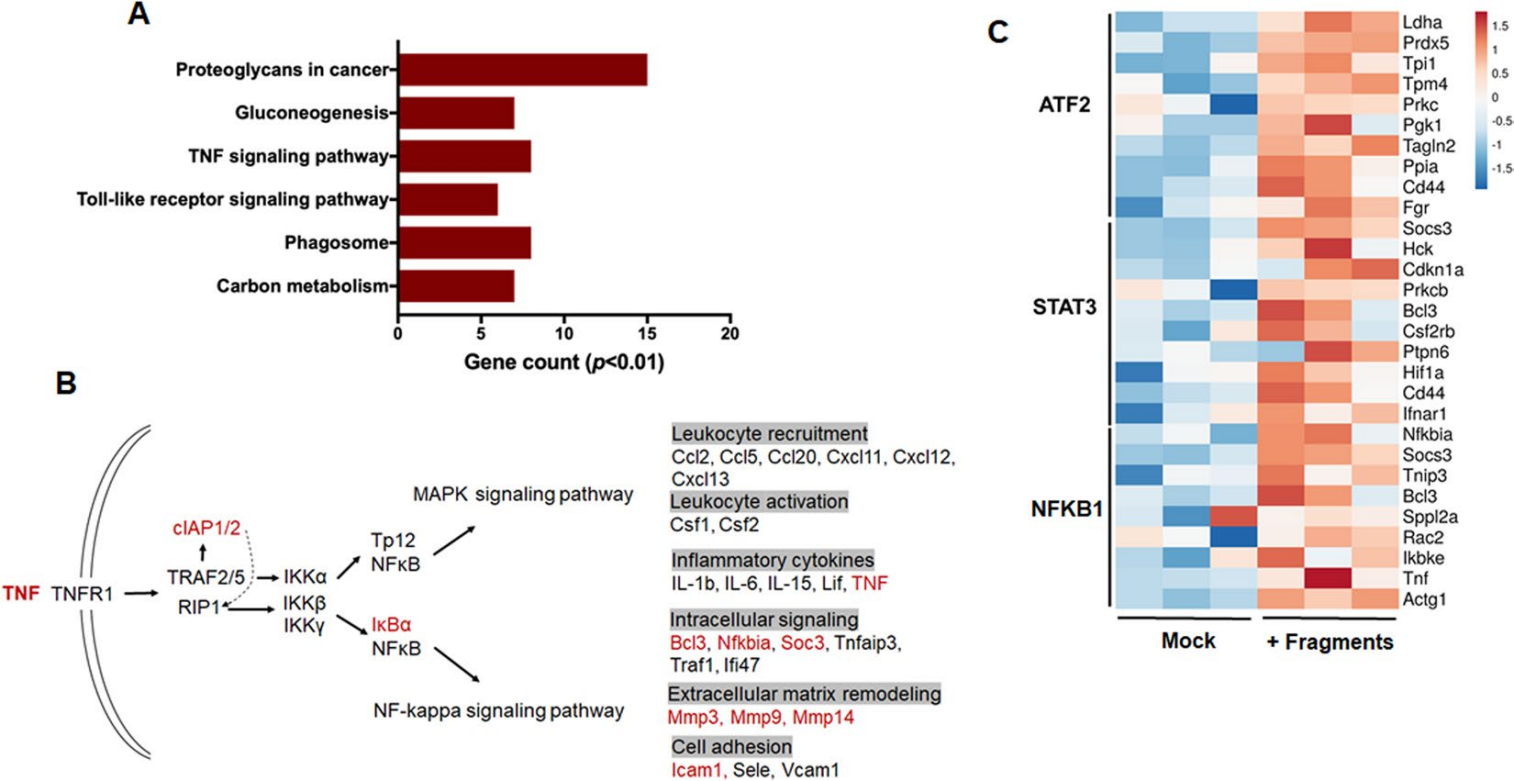
KEGG pathways and transcriptional factors of the differentially expressed genes in macrophages response to cartilage fragments. (**A**) Top enriched pathways. (**B**) TNF signaling pathway generated from KEGG pathway database for the regulated genes in response to cartilage fragments. Red indicates the upregulated genes identified in our study. (**C**) Heat map for the most enriched transcription factors. A scale bar for intense color change from −1.5 or below indicated by blue color or 1.5 or above indicated by red.

To understand the intracellular pathways involved in the response to cartilage fragments, KEGG pathway database mapping analyses were performed. Regulated genes in response to cartilage fragments were enriched in pathways including proteoglycan in cancer, gluconeogenesis, TNF signaling pathway, Toll-like receptor signaling pathway, and phagosome (Fig. 3A,B and Table S6). Likewise, bioinformatic analysis based on Panther and Reactome pathway databases showed that TNF signaling is one of the top terms involved in the response of macrophage to cartilage fragments (data not shown). These results suggest that TNF signaling pathway might be involved in progression of OA. To further gain an insight into the regulation of signaling pathways, transcription factor (TF) enrichment analysis for upregulated genes was performed using TF-protein-protein interactions. The top-enriched transcriptional factors in macrophages exposed to cartilage fragments were ATF2, STAT3 and NFKB1 (Fig. 3C), which are involved in inflammation-related signaling pathways. Taken together, our results demonstrated that interaction between macrophages and cartilage fragments in the synovium may trigger locally chronic inflammatory response associated with development of synovitis. Therefore, reduction of inflammation mediated by macrophages interacting with cartilage fragments might be a therapeutic strategy to control the progression of osteoarthritic process.

### Blockage of macrophage receptors reduced production of TNF-α *in vitro*

**Figure 4:**
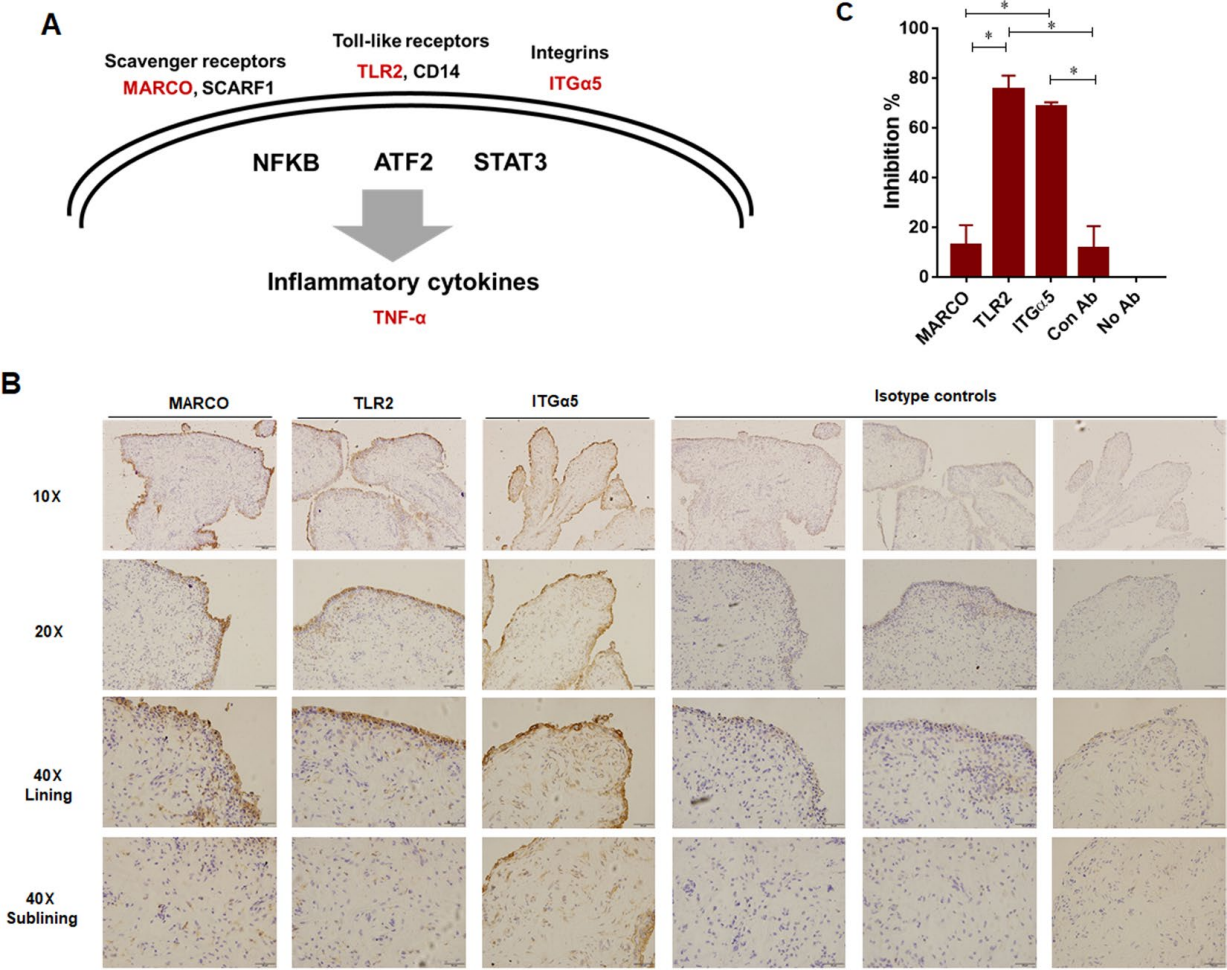
Identification of macrophage receptors to cartilage fragments and functional blocking assay. (**A**) A predicated model of macrophage activation by cartilage fragments based on bioinformatic analysis. (**B**) Detection of MARCO, TLR2 and ITGα5 in synovial tissues of OA patients. Representative images form sections of synovial tissues of 8 OA patients. Lower panels show section stained by rabbit IgG and mouse IgG2 as negative controls. Scale bars are indicated on each image. (**C**) Effects of functional blocking antibodies to MARCO, TLR2 and ITGα5 on the secretion of TNF-α. Macrophages were pretreated with 10 µg/ml for 30 min and then cultured with cartilage fragments. Results are presented as means ± standard errors of the means from 4 wells values for each treatment. Significant difference was determined by one-way ANOVA followed by Tukey’s multiple-comparison procedure. The results are representative of two independent experiments. Experiments were repeated twice for reproducibility of data.

Our bioinformatic analysis suggested that the macrophages stimulated with cartilage fragments expressed number of cell receptors, including MARCO, TLR2, and ITGα5, which might be involved in cell activation (Fig. 4A). Next, we examined the expression of MARCO TLR2, and ITGα5 in the synovial tissues of 8 OA patients using immunohistochemistry. Synovial tissues from all patients showed positive signals mainly in the intima lining layer and only weak signals were observed within sublining layer (Fig. 4B). In contrast, isotype antibodies did not show any reaction (Fig. 4B). These results confirmed our bioinformatic analysis underling the importance of macrophage receptors in inflammatory responses in OA. From these results, we inferred that blockage of these receptors may suppress the inflammatory response and slowdown the progression of OA. To test the hypothesis, macrophages were pretreated with function-blocking antibodies for mouse MARCO, TLR2, and ITGα5 before culturing with cartilage fragments. The production of TNF-α, which is known as major cytokine involved in the physiopathology of OA, was determined in the supernatant of cultures. Preincubation with 10 µg/ml antibody to TLR2 or ITGα5, but not to MARCO resulted in a significant reduction of TNF-α production in macrophage cultures with cartilage fragments (Fig. 4C). In contract, no significant inhibitions were observed at concentration of 1 µg/ml with all types of antibody as well as with higher concentration (20 µg/ml) of antibody to MARCO or SCARF1 (data not shown). There results revealed that TLRs and integrins offer potential targets for therapeutics.

## Discussion

A growing evidence highlights that synovitis is associated with progressive OA and failure of joint, which may suggest the benefits of anti-inflammatory intervention. Addressing the molecular pathways initiating and perpetuating the inflammation in the joint will provide novel therapeutic approach for OA^15,16^. The presence of cartilage fragments in OA joint is closely associated with all stage of disease due to breakage of cartilage under mechanical stress. However, the precise role of cartilage fragments in initiating inflammation and OA process has not been clearly demonstrated. Given the fact that resident macrophages are the major immune cells in synovium encountering the components of damaged cartilage, we studied the gene profiling of macrophages stimulated with cartilage fragments.

Our data demonstrated that macrophages expressed a wide variety of cell-surface receptors in order to recognize and internalize cartilage fragments, including Tlr2, Cd14, Marco, Scarf1, and Itga5. These results are consistent with earlier findings highlighted that TLR2, ITGα5, CD14 and MARCO are strongly expressed in synovial tissues and are involved in progression of OA in experimental models^17–20^. It is evident that components from damaged extracellular matrix, including fibronectin, aggrecan, biglycan, tenascin C, and intracellular proteins of necrotic cells act as danger-associated molecular patterns (DAMPs) that trigger inflammatory responses through activating pattern recognition receptors such as Toll-like receptors and Nod-like receptors^21,22^. Moreover, macrophages can recognize apoptotic cells derived-particles or dying host cell-associated molecules by A scavenger receptors such as SR-A1 and MARCO, class B scavenger receptors such as SR-B1 and CD36, fibronectin receptors such as integrins, Fc receptors such as FcγRIII and complement receptors (CRs) such as CR3, and C-type lectins such as Dectin-1 and Mincle^23^. Activation of these receptors initiates specific intracellular signaling transduction pathways that promote the production of proinflammatory cytokines and the recruitment of leukocytes into the site of injury^21,22^.

Upon phagocytosis of damaged tissues by macrophages, the undigested materials trigger lysosomal damage followed by inflammasome activation and production of proinflammatory cytokines including IL-1β and IL-18. In a related study, Jin *et al*., showed that hydroxyapatite crystals found in OA joints simulate robust secretion of macrophages IL-1β and IL-18 in a NLRP3 inflammasome-dependent manner^24^. However, our data showed that IL-1β was not upregulated in macrophages stimulated with cartilage fragments. In consistent with our data, a recent study showed that Il-1β, and Nlrp3 inflammasome are not key mediators in murine menisectomy model of osteoarthritis^25^. On the other hand, macrophages stimulated with cartilage fragments upregulated number of genes that are predominantly expressed in synovitis of OA patients such as TNF, CCL9, CXCL16, and PTGES^15,16,21,26–29^. In line with our findings, damaged extracellular matrix-derived molecules including biglycan, decorin, versican, tenascin-C, fibrinogen and hyaluronic are known to elicit a sterile proinflammatory response in macrophages via interacting with TLR2 and TLR4. The interaction between these molecules and TLR2, 4 leads to activation of adaptor molecule myeloid differentiation primary response gene 88 (MyD88) resulting in production of proinflammatory mediators including TNF-α, IL-1β, CXCL1, CXCL2, CCL2^30^. In addition, MyD88-dependent TLR signaling activates IRAK family of kinases, which in turn stimulate the E3 ubiquitin ligase activity of TRAF6 and transforming growth factor-β-activated kinase 1 (TAK1) resulting in NF-κB activation^31^.

Another positive regulator of inflammatory response found in the gene profile was Aif1, which is known to be highly expressed in synovial tissues of rheumatoid arthritis patients^32^. In addition, macrophages in response to cartilage fragments stimulation expressed proteolytic enzymes including matrix metalloproteinases Mmp9 and Mmp14, and disintegrin and metalloproteinase domain-containing protein 17 (Adam17), which are known to be involved in demineralization of bone, degradation of components of the extracellular matrix and digestion of matrix proteins^33–36^. Not surprisingly, our bioinformatic analysis revealed that the upregulated genes in stimulated macrophages were significantly enriched in rheumatoid arthritis. Together, these results reveal that macrophages elicited both inflammatory and catabolic molecules in response to cartilage fragments that may reflect the pathogenesis of osteoarthritis. Our future research includes studying the transcriptional profile of chondrocytes co-cultured with stimulated macrophages by cartilage fragments in order to understand the molecular mechanism associated with cartilage degeneration.

The most important findings in our study is that cartilage fragments activated TNF signaling pathway through nuclear factor-kappa B (NF-κB) intracellular signaling pathway. NF-κB pathway is crucial for development of synovitis features of the OA joint, and thus has been documented as a promising therapeutic target^37^. In fact, NF-κB pathway play a key regulatory role in both stress and inflammatory in OA through regulating other transcription factors, including E74-like factor 3 (ELF3) and HIF-2α. This leads to an upregulation of IL-8, IL-1β, IL-6, TNF-α and receptor activator of nuclear factor kappa-B ligand (RANKL), cyclooxygenase 2 (COX2) and angiogenic factor of vascular endothelial growth factor (VEGF) in the synovium^38^. Our further results showed that blockage of TLR2 and ITGα5 but not scavenger receptors (MARCO or SCARF1) inhibited the production of TNF-α of macrophages in response to cartilage fragments. These results are consistent with earlier studies documenting that TLR2 is upregulated in the synovial tissue from OA patients and is involved in the pathogenesis of osteoarthritis^39–41^. Signaling via TLRs leads to a rapid activation of NF-κB associated with the elevated expression of proinflammatory cytokines and synovial macrophage^25^. Likewise, ITGα5 is the most promiscuous member of integrin that interacts with variety of proteins such as vitronectin, fibronectin, and thrombospondin and is known to mediate a vascular development inflammation and matrix degradation in OA^17,42^. ITGα5 can promote an NF-κB signaling pathway resulting in a unique gene expression program involved in inflammation^43^. On the other hand, the inability of MARCO antibody to inhibit TNF-α production can be explained by the fact that scavenger receptors limit production of proinflammatory cytokines of innate immune cells in response to bacterial and fungal pathogens as well as crystalline silica particles^44,45^. Taken together, macrophages exposed to cartilage fragments elicited common gene expression signatures for synovitis and inflammation in the joint. Continuing research on TLR2 and ITGα5 as therapeutic targets may aid in designing novel medications for osteoarthritis.

The major limitations in the current study include inability to use human samples in this cartilage fragments stimulation model. In fact, we were not able to prepare cartilage fragments from patients undergoing knee arthroplasty due to the damaged or inflamed condition of cartilage tissue in the patients. Moreover, the macrophages used in the current study were bone marrow-derived macrophages, which might exhibit different molecular responses from synovial resident macrophages. Our future study includes analyzing gene profiling of human synovial macrophage phagocytizing cartilage fragments (only TLR2-positive macrophages) to gain the precise molecular responses to cartilage fragments. One more limitation is the lack of *in vivo* data of inhibitory assay using blocking antibodies in animal models. In fact, surgically induced-animal models do not produce enough cartilage fragments in the joint and seem to be not suitable for confirming our findings.

In conclusion, we report fundamental knowledge regarding the molecular responses of macrophages to cartilage fragments. Our data provide a new insight into the molecular pathogenesis of osteoarthritis and shed light on new molecular candidates for therapeutic intervention and diagnostic applications.

## Methods

### Ethics statement. Ethics statement

Our study was conducted according to the protocol guidelines of Hokkaido University and approved by the Research Ethics Review Committee of Hokkaido University. All procedures for animal experiments were performed based on the ethical guidelines approved by the animal care committee of Hokkaido University. (approval ID:17-0085). Our research protocols for human samples used in this study was approved by the Research Ethics Review Committee of Hokkaido University Hospital (approval ID: 016-0177). Informed consents for the use of samples in our research were obtained from all donors.

### Preparation of cartilage fragments and culture with macrophages

Cartilage fragments and murine macrophages were prepared and cultured as described in our earlier study^12^. Briefly, cartilages were isolated from femoral head cartilages of 4-week-old wild type C57BL/6 male mice and then crushed by Multi Beads Shocker (Yasui Kikai, Osaka, Japan) for 1 minute at 2500 rpm. Fragments were washed twice using ice-cold phosphate-buffered saline buffer (PBS; Nacalai tesque, Kyoto, Japan) and subjected to a particle image analyzer Morphologi G3 (Malvern Instruments, Malvern, UK) and scanning electron microscope (SEM):S-4800 (Hitachi High-Technologies Corporation, Tokyo, Japan) for examining their sizes, shapes and surface topography. Endotoxins in the suspended PBS-cartilage fragments were determined using ToxinSensor Single Test Kit (GenScript, Piscataway, USA). Prepared cartilage fragments had sizes (0.54 to 55μm with a mean of 3.11μm), shapes and surface topography similar to those found in patients with osteoarthritis^14^. Endotoxins were below the detection limit of kit (0.015 EU/ml) in all tested samples. Bone marrow cells (BMC) were isolated from the same mice sacrificed for cartilage fragments and added to monocyte isolation kit BM (Miltenyi Biotec, Bergisch Gladbach, Germany). Cells were then cultured in RPMI-1640 with 25 mg/l penicillin/streptomycin and 10% heat-inactivated fetal bovine serum (Sigma-Aldrich, St. Louis, USA) supplemented with 50 ng/ml mouse recombinant macrophage colony-stimulating factor (Mcsf; PeproTech, Rocky Hill, USA) for 7 days. Thereafter, differentiated macrophages were detached and seed in 24-well-plates at 2×10^5^ cells/well. Moreover, thioglycolate (Sigma-Aldrich)-elicited peritoneal macrophages were harvested in PBS, washed and seed in 24-well-plates at 2×10^5^ cells/well. Macrophages were cultured for 2 h in RPMI-1640 supplemented with 25 mg/l penicillin/streptomycin and 10% heat-inactivated fetal bovine serum and attached cells were washed by PBS for further stimulation. Cartilage fragments were resuspended in medium and added to macrophage cultures at ratio of 5:1 for a cultivation period of 24 h.

### RNA isolation, library generation and sequencing

Differentiated macrophages cultured with or without cartilage fragments were lysed with TRIzol Reagent (Invitrogen, Carlsbad, USA) and harvested for RNA purification. RNA was purified using RNeasy Plus Mini kit (QIAGEN, Hilden, Germany) according to the manufacturer’s instructions, and integrity of each RNA sample was assessed by determining 28 S/18 S ribosomal RNA bands with an Agilent 2100 bioanalyzer (Agilent Technologies, Santa Clara, USA). High-quality DNA-free RNA with integrity score> 9.0 was used to generate libraries using the TruSeq Stranded mRNA Sample Preparation Kit (Illumina, San Diego, USA) and their quality was examined by Bioanalyzer High sensitivity DNA kit (Agilent Technologies). The paired-end reads (100 bp) were further obtained by Illumina HiSeq. 2500 (Illumina). 60 million reads per sample were mapped by alignment to mouse genome (mm10) using TopHat and Bowtie, normalized using the Trimmed Mean of M values (TMM) process, and then annotated using Cufflinks and Cuffdiff (http://cole-trapnell-lab.github.io/cufflinks/cuffdiff/index.html). Low calculated fragments per kilobase of transcript per million mapped reads (FPKM) were removed^46^. The RNA-seq data are publicly available at the Gene Expression Omnibus (GEO) database (http: www.ncbi.nlm.nih.-gov/geo/) under the accession number GSE141308.

### Bioinformatics analysis

Significantly expressed genes with p-value <0.05 were subjected to gene ontology (GO) enrichment analyses, enrichment analyses of KEGG (Kyoto Encyclopedia of Genes and Genome) pathway^47^ and PANTHER and Reactome databases (http://geneontology.org/). The Database for Annotation Visualization and Integrated Discovery online tools (DAVID: https://david.ncifcrf.gov/) was used for analyses. The cut-off values for statistically significant differences of each enriched term were gene count>4 and p < 0.001. To confirm the results of the gene enrichment, further analyses were carried out by using Gene Ranker (http://www.genomatix.de/) and Toppgene (https://toppgene.cchmc.org/enrichment.jsp). The differences in fold changes were visualized by heat map (http://biit.cs.ut.ee/clustvis/).

### Quantitative real-time reverse transcription polymerase chain reaction (qRT–PCR)

First-strand cDNA was synthesized from purified RNA (0.5 µg) of each sample using GoScript Reverse Transcriptase kit (Promega, Madison, USA) to cDNA. The qRT-PCR was performed using SYBR_ Premix Ex Taq II (Takara, Shiga, Japan) with specific primers (Table S7) on a Thermal Cycler Dice System 2 (Takara). Gene expression of target gene was calculated after normalizing to the expression of GAPDH housekeeping gene using the 2^ΔΔCT^ method.

### Human synovial tissues and immunohistochemistry

Synovial tissue samples were collected from the suprapatellar pouch of 8 OA patients (5 women and 3 men; mean age 75 years, range 53–84) undergoing total knee arthroplasty. All OA cases were diagnosed according to clinical features and X-ray imaging and they were Kellgren and Lawrence grade IV. Tissues from inflamed synovial membranes were chosen according to standardized macroscopic criteria established by Ayral^48^. Obtained tissues were embedded in Tissue-Tek OCT compound (Sakura Finetek Japan, Tokyo, Japan) and snap-frozen in liquid nitrogen. Thereafter, frozen tissues were sectioned (5 µM) were fixed with actetone for 10 minutes, air-dried, and blocked with 0.3% H2O2 for 10 minutes to prevent endogenous peroxidase reactivity. The slides were blocked with PBS-containing 1% bovine serum albumin plus 5% horse serum for 1 hour and incubated with primary antibodies including 1:50 monoclonal mouse anti-human TLR2 (Toll-like receptor 2) (Abcam, Cambridge, UK), 1:250 polyclonal rabbit anti-human MARCO (macrophage receptor with collagenous structure) (Atlas Antibodies, Stockholm, Sweden) and 1:100 monoclonal rabbit anti-human ITGα5 (integrin alpha 5) (Abcam) overnight at 4 °C. After washing with PBS-0.05% Tween (Wako, Osaka, Japan), slides were incubated for 30 minutes with each respective secondary antibody (Abcam). Signal was amplified with horseradish peroxidase (HRP)-conjugated streptavidin using a Vectastain Elite ABC kit (Vector Laboratories, Burlingame, USA) followed by counterstaining with hematoxylin. Staining specificity was confirmed by utilizing isotype-matched immunoglobulin controls including mouse IgG2a and rabbit IgG (Abcam).

### Antibody blocking assay

Functional blocking antibody of each target cell receptor was added to macrophage cultures to inhibit their interaction with cartilage fragments. Antibodies included anti-TLR2 (InvivoGen, San Diego, USA), anti-MARCO (Serotec, Hercules, USA), anti-ITGα5 (Millipore, Burlington, USA) and mouse IgGs (GeneTex, Irvine, USA) used as negative control. Bone marrow-derived macrophages were cultured in medium supplemented with different blocking antibodies at concentrations of 1 or 10 μg/ml for 30 min. Thereafter, cells were washed twice with ice-cold PBS and cultured with cartilage fragments at ratio of 1:5 for 24 h. Stimulation with lipopolysaccharides (LPS; 50 ng/mL Sigma-Aldrich) was used as positive control to verify the condition of experiment. Supernatant of cultures was harvested for detection of TNF-α by enzyme linked immunosorbent assay (ELISA). Assay was preformed according to the manufacturer’s instructions: mouse TNF-α detection ELISA kit (R&D Systems, Minneapolis, USA).

### Statistical analysis

The false discovery rate (FDR) control method with a threshold of p-value <0.05 by multiple tests were used to determine the significant differences in transcripts between macrophages cultured with cartilage fragments and cultured alone (mock). A gene was considered significantly differentially expressed when its FDR corrected p-value (q-value) was <0.05. Student’s t test was performed to compare the results of the gene expression examined by qRT-PCR. One-way analysis of variance (ANOVA) followed by Tukey’s multiple-comparison procedure was performed to evaluate the differences among groups treated with antibodies. Results were presented as means ± standard errors of the means (SEM) and considered statistically significant at *p* ≤ 0.05 (GraphPad, San Diego, USA).

## Supplementary information

Supplementary Information.
