# Supplementary material for: Transcriptional profiling of murine macrophages stimulated with cartilage fragments revealed a strategy for treatment of progressive osteoarthritis
Source: Sci Rep. 2020 May 5;10:7558. doi: 10.1038/s41598-020-64515-1 (PMC7200748; doi:10.1038/s41598-020-64515-1)
Supplement: Supplementary file 1 — Supplementary Information. [file 41598_2020_64515_MOESM1_ESM.docx]

**Transcriptional profiling of murine macrophages stimulated with cartilage fragments revealed a strategy for treatment of progressive osteoarthritis**

Masanari Hamasaki^1^, Mohamad Alaa Terkawi^1, 2^, Tomohiro Onodera^1, 2^, Yuan Tian^1^, Taku Ebata^1^, Gen Matsumae^1^, Hend Alhasan^1^, Daisuke Takahashi^1^, Norimasa Iwasaki^1, 2^

^1^Department of Orthopedic Surgery, Faculty of Medicine and Graduate School of Medicine, Hokkaido University, Kita-15, Nish-7, Kita-ku, Sapporo, 060-8638, Japan.

^2^Global Institution for Collaborative Research and Education (GI-CoRE). Frontier Research Center for Advanced Material and Life Science Bldg No 2. Hokkaido University.

^*^**Corresponding authors**

Mohamad Alaa Terkawi, DVM, PhD, Department of Orthopaedic Surgery, Faculty of Medicine and Graduate School of Medicine, Hokkaido University, Kita-15, Nishi-7, Kita-ku, Sapporo, Japan. Telephone: +81-11-706-5935, Fax: +81-11-706-6054.

E-mail: [materkawi@med.hokudai.ac.jp](mailto:materkawi@med.hokudai.ac.jp)

Tomohiro Onodera, MD, PhD, Department of Orthopaedic Surgery, Faculty of Medicine and Graduate School of Medicine, Hokkaido University, Kita-15, Nishi-7, Kita-ku, Sapporo, Japan. Telephone: +81-11-706-5935, Fax: +81-11-706-6054.

E-mail: [tomozou@med.hokudai.ac.jp](mailto:tomozou@med.hokudai.ac.jp)

**Supplementary information**

**Table S1.** Significantly up- and down-regulated genes in macrophages stimulated with cartilage fragments.

| Gene Symbol | Gene name (or Protein name) | p value | q value | log2 FC |
| --- | --- | --- | --- | --- |
| Marco  Ptges  Saa3  Irg1  Hp  Fpr2  Clec4e  Pilra  Slpi  Ly6c2  Socs3  Tnf  Fpr1  Slc2a6  Acsl1  Tarm1  C3  Mmp9  Ehd1  Sod2  Cav1  Marcksl1  Slc31a2  Il2rg  Irak3  Pf4  Aoah  Arl5c  Lpcat2  Mfsd7a  Mmp14  Aif1  Glrx  Nck1  Myadm  Tnip3  Ccdc86  Bst1  Tgfbi  Bcl3  Msmo1  Aldoc  Nlrp3  Pfkl  Fam102b  Prdx5  Ikbke  Abracl  Sdc4  Hcls1  Pid1  Lilr4b  Lst1  Kcnn4  Fgr  Hif1a  Scarf1  Cpd  Ctsc  Icam1  C1qa  Dnajc10  Slc16a10  Cd14  Hsd17b12  Gaa  N4bp1  Lcp2  Tagln2  Sdc1  Birc3  Wfdc17  Bst2  Ptgir  Hck  Adgre1  Acpp  Nfkbia  Cfp  B4galt6  Acp2  Gmfg  Nadk  Rhoq  Pbxip1  Atp5h  Cdkn1a  Tgm2  Dock7  Pgap2  Txn1  Cd81  Cd52  Cyfip1  Plek  Ptpn7  Slc25a37  Tlr2  Ifnar1  Tpm4  Anxa3  Igsf6  Cd302  Pirb  Slfn2  Cmklr1  Itga5  Lars2  Neat1  Gnas  Rab10  Atox1  Pgk1  Mapkapk2  Ccl9  Ankrd13a  Slc31a1  Rn45s  Slfn5  Lgals3bp  Arl5a  Flna  Actg1  Sppl2a  Cxcl16  Gapdh  Tpi1  Alas1  Rac2  Adam17  Cd44  Rassf4  Ldha  Clptm1  Ptpn6  Fam20c  Mvp  Prkcb  Cybb  Tmsb4x  Prdx1  Rhog  Esd  Ms4a7  Cstb  M6pr  Ptprj  Rnf149  Tnfaip2  Ppia  C1qb  Csf2rb  Gpi1 | Macrophage receptor with collagenous structure  Prostaglandin E synthase  Serum amyloid A-3  Aconitate decarboxylase  Haptoglobin  Formyl peptide receptor 2  C-type lectin domain family 4 member E  Paired immunoglobulin-like type 2 receptor alpha  Secretory leukocyte protease inhibitor  Lymphocyte antigen 6C2  Suppressor of cytokine signaling 3  Tumor necrosis factor  N-formyl peptide receptor  Solute carrier family 2, facilitated glucose transporter member 6  Long-chain-fatty-acid--CoA ligase 1)  T-cell-interacting,-activating receptor on myeloid cells protein 1  Complement C3  Matrix metalloproteinase-9  EH domain-containing protein 1  Superoxide dismutase  Caveolin-1  Macrophage myristoylated alanine-rich C kinase substrate  Solute carrier family 31 member 2  Interleukin-2 receptor subunit gamma  Interleukin-1 receptor-associated kinase 3  Platelet factor 4  Acyloxyacyl hydrolase  ADP-ribosylation factor-like protein 5C  Lysophosphatidylcholine acyltransferase 2  Major facilitator superfamily domain containing 7A  Matrix metalloproteinase-14  Allograft inflammatory factor 1  Glutaredoxin-1  NCK adaptor protein 1  Myeloid-associated differentiation marker  TNFAIP3-interacting protein 3  Coiled-coil domain-containing protein 86  Bone marrow stromal antigen 1  Transforming growth factor, beta induced  B-cell lymphoma 3 protein homolog  Methylsterol monooxygenase 1  Fructose-bisphosphate aldolase C  NACHT, LRR and PYD domains-containing protein 3  ATP-dependent 6-phosphofructokinase, liver type  Protein FAM102B  Peroxiredoxin-5, mitochondrial  Inhibitor of nuclear factor kappa-B kinase subunit epsilon  ABRA C-terminal-like protein  Syndecan-4  Hematopoietic cell-specific LYN substrate 1  Phosphotyrosine interaction domain-containing protein 1  Leukocyte immunoglobulin-like receptor, subfamily B, member 4B  Leukocyte-specific transcript 1 protein  Intermediate conductance calcium-activated potassium channel protein 4  Tyrosine-protein kinase Fgr  Hypoxia-inducible factor 1-alpha  Scavenger Receptor Class F Member 1  Carboxypeptidase D  Cathepsin C  Intercellular adhesion molecule 1  Complement C1q subcomponent subunit A  DnaJ homolog subfamily C member 10  Solute carrier family 16 member 10  Monocyte differentiation antigen CD14  17-beta-hydroxysteroid dehydrogenase 12  Lysosomal alpha-glucosidase  NEDD4-binding protein 1  Lymphocyte cytosolic protein 2  Transgelin-2  Syndecan-1  Baculoviral IAP repeat-containing protein 3  WAP four-disulfide core domain 17  Bone marrow stromal antigen 2  Prostaglandin I2 receptor  Tyrosine-protein kinase HCK  Adhesion G Protein-Coupled Receptor E1  Prostatic acid phosphatase  NF-kappa-B inhibitor alpha  Complement factor P  Beta-1,4-galactosyltransferase 6  Acid Phosphatase 2, Lysosomal  Glia maturation factor  NAD kinase  Rho-related GTP-binding protein RhoQ  Pre-B-cell leukemia transcription factor-interacting protein 1  ATP synthase, H+-transporting, mitochondrial F0 complex, subunit D  Cyclin-dependent kinase inhibitor 1  Transglutaminase-2  Dedicator of cytokinesis protein 7  Post-GPI attachment to proteins factor 2  Thioredoxin  CD81 antigen  CD52 antigen  Cytoplasmic FMR1-interacting protein 1  Pleckstrin  Tyrosine-protein phosphatase non-receptor type 7  Solute carrier family 25 member 37  Toll-like receptor 2  Interferon alpha/beta receptor 1  Tropomyosin-4Homo sapiens serum/glucocorticoid regulated kinase (SGK)  Annexin A3  Immunoglobulin superfamily member 6  CD302 antigen  Paired immunoglobulin-like receptor B  Schlafen 2  Chemokine-like receptor 1  Integrin alpha-5  Leucyl-tRNA synthetase  Nuclear Paraspeckle Assembly Transcript 1  Guanine nucleotide-binding protein G(s) subunit alpha isoforms short  Ras-related protein Rab-10  Copper transport protein ATOX1  Phosphoglycerate kinase 1  MAP kinase-activated protein kinase 2  C-C motif chemokine 9  Ankyrin repeat domain-containing protein 13A  Solute carrier family 31 member 1  45S pre-ribosomal RNA  Schlafen family member 5  Lectin galactoside-binding soluble 3-binding protein  ADP-ribosylation factor-like protein 5A  Filamin-A  Actin, cytoplasmic 2  Signal peptide peptidase-like 2A  C-X-C motif chemokine 16  Glyceraldehyde-3-phosphate dehydrogenase  Triosephosphate isomerase  Delta-aminolevulinate synthase 1  Ras-related C3 botulinum toxin substrate 2  Disintegrin and metalloproteinase domain-containing protein 17  CD44 antigen  Ras association domain-containing protein 4  L-lactate dehydrogenase A chain  Cleft lip and palate transmembrane protein 1 homolog  Tyrosine-protein phosphatase non-receptor type 6  Extracellular serine/threonine protein kinase FAM20C  Major vault protein  Protein kinase C beta type  Cytochrome b(558) subunit beta  Thymosin beta-4  Peroxiredoxin-1  Rho-related GTP-binding protein RhoG  Esterase D  Membrane-spanning 4-domains, subfamily A, member 7  Cystatin-B  Cation-dependent mannose-6-phosphate receptor  Protein tyrosine phosphatase  RING finger protein 149  Tumor necrosis factor alpha-induced protein 2  Peptidyl-prolyl cis-trans isomerase A  Complement C1q subcomponent subunit B  Cytokine receptor common subunit beta  Glucose-6-phosphate isomerase | 5.00E-05  0.00065  5.00E-05  5.00E-05  5.00E-05  5.00E-05  5.00E-05  5.00E-05  5.00E-05  5.00E-05  0.0001  5.00E-05  5.00E-05  0.0015  0.0009  0.00115  5.00E-05  5.00E-05  5.00E-05  5.00E-05  5.00E-05  5.00E-05  0.0009  5.00E-05  5.00E-05  5.00E-05  5.00E-05  5.00E-05  5.00E-05  0.00215  5.00E-05  0.0004  5.00E-05  0.0023  5.00E-05  0.00015  0.0016  5.00E-05  5.00E-05  0.00105  0.00035  0.0014  5.00E-05  5.00E-05  5.00E-05  5.00E-05  5.00E-05  0.0001  5.00E-05  5.00E-05  5.00E-05  5.00E-05  0.00065  0.0013  5.00E-05  5.00E-05  0.00225  5.00E-05  5.00E-05  5.00E-05  5.00E-05  5.00E-05  5.00E-05  5.00E-05  0.00065  0.00015  5.00E-05  0.00105  5.00E-05  0.0004  0.0001  0.00145  0.0027  0.0012  5.00E-05  5.00E-05  0.0027  0.00095  5.00E-05  0.00035  5.00E-05  0.0006  5.00E-05  0.0007  0.0002  0.0003  0.0004  5.00E-05  0.0009  0.001  5.00E-05  0.00095  5.00E-05  5.00E-05  5.00E-05  0.00275  0.00195  5.00E-05  0.00055  5.00E-05  5.00E-05  5.00E-05  0.0007  5.00E-05  5.00E-05  0.0013  0.0014  0.00045  5.00E-05  0.0018  0.0017  0.00055  0.0002  5.00E-05  5.00E-05  0.00125  0.0002  5.00E-05  0.0007  5.00E-05  0.0006  5.00E-05  0.00015  0.0006  5.00E-05  5.00E-05  0.0004  0.0001  0.0009  0.0007  0.00045  5.00E-05  5.00E-05  0.0018  0.0011  0.0002  0.00105  0.0016  0.0022  0.0016  0.00105  0.0013  0.0003  0.0012  0.0003  0.0016  0.0008  0.0009  5.00E-05  0.00015  0.00275  0.00195  0.00085 | 0.00185882  0.0162463  0.00185882  0.00185882  0.00185882  0.00185882  0.00185882  0.00185882  0.00185882  0.00185882  0.00342946  0.00185882  0.00185882  0.0297578  0.0207375  0.0248176  0.00185882  0.00185882  0.00185882  0.00185882  0.00185882  0.00185882  0.0207375  0.00185882  0.00185882  0.00185882  0.00185882  0.00185882  0.00185882  0.0397975  0.00185882  0.0112  0.00185882  0.0418733  0.00185882  0.0048087  0.0310456  0.00185882  0.00185882  0.022996  0.0100545  0.028411  0.00185882  0.00185882  0.00185882  0.00185882  0.00185882  0.00342946  0.00185882  0.00185882  0.00185882  0.00185882  0.0162463  0.0268748  0.00185882  0.00185882  0.0413029  0.00185882  0.00185882  0.00185882  0.00185882  0.00185882  0.00185882  0.00185882  0.0162463  0.0048087  0.00185882  0.022996  0.00185882  0.0112  0.00342946  0.0292913  0.0474  0.0256464  0.00185882  0.00185882  0.0474  0.0215528  0.00185882  0.0100545  0.00185882  0.0152552  0.00185882  0.0171094  0.00610207  0.008848  0.0112  0.00185882  0.0207375  0.0225714  0.00185882  0.0215528  0.00185882  0.00185882  0.00185882  0.0475234  0.0368667  0.00185882  0.0144833  0.00185882  0.00185882  0.00185882  0.0171094  0.00185882  0.00185882  0.0268748  0.028411  0.012139  0.00185882  0.0343241  0.0328419  0.0144833  0.00610207  0.00185882  0.00185882  0.0264593  0.00610207  0.00185882  0.0171094  0.00185882  0.0152552  0.00185882  0.0048087  0.0152552  0.00185882  0.00185882  0.0112  0.00342946  0.0207375  0.0171094  0.012139  0.00185882  0.00185882  0.0343241  0.0238549  0.00610207  0.022996  0.0310456  0.0405533  0.0310456  0.022996  0.0268748  0.008848  0.0256464  0.008848  0.0310456  0.0192348  0.0207375  0.00185882  0.0048087  0.0475234  0.0368667  0.0203265 | 4.00031  3.54416  3.04337  2.79298  2.69704  2.64041  2.47904  2.23334  2.01779  2.01123  2.00014  1.83508  1.75039  1.66082  1.43837  1.37277  1.35983  1.29723  1.26873  1.26497  1.22344  1.1225  1.08585  1.08246  1.0418  1.03253  1.01809  1.00782  0.994342  0.983482  0.969651  0.952248  0.929867  0.887815  0.871449  0.839728  0.813832  0.803258  0.796297  0.790767  0.787692  0.784401  0.776288  0.766728  0.740974  0.736843  0.728374  0.726584  0.726385  0.720546  0.707453  0.695609  0.692447  0.684974  0.678868  0.668229  0.664026  0.663238  0.662411  0.633389  0.628256  0.622431  0.62088  0.616524  0.608286  0.607326  0.604961  0.604471  0.598803  0.597561  0.59717  0.584371  0.583447  0.580104  0.568376  0.556398  0.554664  0.547943  0.541898  0.534183  0.531364  0.530048  0.529918  0.529514  0.521577  0.517658  0.507803  0.503631  0.490451  0.485565  0.484151  0.484123  0.477256  0.471709  0.471237  0.464276  0.463692  0.463624  0.456763  0.447825  0.445369  0.440532  0.433302  0.429471  0.419136  0.408576  0.408204  0.407754  0.407099  0.391637  0.381256  0.378278  0.378223  0.373767  0.366896  0.365581  0.363919  0.360247  0.357263  0.357141  0.353596  0.349693  0.333524  0.332826  0.330703  0.329672  0.327249  0.319455  0.314465  0.313789  0.304816  0.304088  0.302469  0.292953  0.281826  0.278431  0.271219  0.265919  0.265606  0.26552  0.261165  0.244358  0.241583  0.234782  0.233551  0.23052  0.225637  0.216819  0.215478  0.195496  0.189806  0.188311  0.177229 |
| Fcgr3  Atp6v1a  Soat1  Atp6v1b2  Stom  Malat1  Anpep  Cd93  Nceh1  Fnip2  Slc6a6  Pld3  Npc1  Cd300a  Rab8b  Zfp36l2  Man2b1  Myo5a  Creg1  Dennd4c  Por  Rassf2  Lgmn  Fam129b  Xdh  Dusp3  Gpnmb  Tns3  Pnpla7  Plxnc1  Rsrp1  Plin2  Tmem189  Emp1  Fabp5  Ccl6  Tpd52  H2afz  Tbk1  Herc1  Atp1b3  Man2a2  Ptpra  BC005537  Mmp8  Slc37a2  Idh1  Mmp12  Rhob  Id3  Cebpg  Piezo1  Atp6v0d2  Antxr2  Alcam  Mroh1  Col1a2  Cd300lb  Id2  Il7r  Golga4  Colec12  Sat1  Tfrc  Man1c1  Ncoa3  Tlr4  Bckdha  Apoe  Arl4c  Lrp12  Col5a2  Kif3a  St8sia4  Gas6  Abcg1  Hpgds  Tlr8  Engase  Mfge8  H2-Aa  Ier5  Capn7  Zfp36l1  Zdhhc14  Lpxn  Arhgef6  H2-Ab1  Hsd17b4  Slc38a2  Slc9a9  Ccny  Col1a1  Mamdc2  Tm6sf1  Lilr4b  Slc6a12  H2-Eb1  Cd74  Cd28  Osbpl8  Api5  Ogt  Cnot1  Slco4a1 | Fc-gamma RIII  V-type proton ATPase catalytic subunit A  Sterol O-acyltransferase 1  Vacuolar proton pump subunit B  Stomatin  Metastasis associated lung adenocarcinoma transcript 1  Aminopeptidase N  Complement component C1q receptor  Neutral cholesterol ester hydrolase 1  Folliculin-interacting protein 2  Solute carrier family 6 member 6  Phospholipase D3  Niemann-Pick C1 protein  CD300 antigen-like family member A  RAB8B, member RAS oncogene family  Zinc finger protein 36, C3H1 type-like 2  Mannosidase alpha class 2B member 1  Unconventional myosin-Va  Cellular repressor of E1A-stimulated genes 1  DENN domain-containing protein 4C  Cytochrome P450 Oxidoreductase  Ras association domain-containing protein 2  Legumain  Family with sequence similarity 129, member B  Xanthine dehydrogenase/oxidase  Dual specificity protein phosphatase 3  Transmembrane glycoprotein NMB  Tensin-3  Patatin-like phospholipase domain-containing protein 7  Plexin-C1  Arginine/serine-rich protein 1  Perilipin-2  Transmembrane protein 189  Epithelial membrane protein 1  Fatty acid-binding protein 5  C-C motif chemokine 6  Tumor protein D52  Histone H2A.Z  TANK-binding kinase 1  HECT And RLD Domain Containing E3 Ubiquitin Protein Ligase Family Member1　Sodium/potassium-dependent ATPase subunit beta-3  Mannosidase alpha class 2A member 2  Receptor-type tyrosine-protein phosphatase alpha  [BC005537](http://www.informatics.jax.org/marker/MGI:2441726)  Matrix metalloproteinase-8  Solute carrier family 37 member 2  Isocitrate dehydrogenase [NADP] cytoplasmic  Matrix metalloproteinase-12  Rho-related GTP-binding protein RhoB  Inhibitor of DNA binding 3  CCAAT/enhancer-binding protein gamma  Piezo-type mechanosensitive ion channel component 1  V-type proton ATPase subunit d 2  Anthrax toxin receptor 2  Activated leukocyte cell adhesion molecule  MKIAA1833 protein  Collagen alpha-2(I) chain  CD300 antigen-like family member B  Inhibitor of DNA binding 2  Interleukin-7 receptor subunit alpha  Golgin subfamily A member 4  Collectin-12  Spermidine/spermine N(1)-acetyltransferase 1  Transferrin receptor protein 1  alpha-1,2-Mannosidase  Nuclear receptor coactivator 3  Toll-like receptor 4  Branched-chain alpha-keto acid dehydrogenase E1 component alpha chain  Apolipoprotein E  ADP-ribosylation factor-like protein 4C  Low-density lipoprotein receptor-related protein 12  Collagen alpha-2(V) chain  Kinesin-like protein KIF3A  ST8 Alpha-N-Acetyl-Neuraminide Alpha-2,8-Sialyltransferase 4  Growth arrest-specific protein 6  ATP-binding cassette sub-family G member 1  Hematopoietic prostaglandin D synthase)  Toll-like receptor 8  Cytosolic endo-beta-N-acetylglucosaminidase  Milk fat globule-EGF factor 8  H-2 class II histocompatibility antigen, A-B alpha chain  Immediate early response gene 5 protein  Calpain-7  Zinc finger protein 36, C3H1 type-like 1  Zinc finger DHHC domain-containing protein 14  Leupaxin  Rho guanine nucleotide exchange factor 6  H-2 class II histocompatibility antigen, A beta chain  17-beta-hydroxysteroid dehydrogenase 4  Solute carrier family 38 member 2  Solute carrier family 9 member 9  Cyclin fold protein 1  Collagen alpha-1(I) chain  MAM domain-containing protein 2  Transmembrane 6 superfamily member 1  Leukocyte immunoglobulin-like receptor, subfamily B, member 4B  Solute carrier family 6 member 12  H-2 class II histocompatibility antigen, I-E beta chain  CD_antigen: CD74  CD_antigen: CD28  Oxysterol-binding protein-related protein 8  Apoptosis inhibitor 5  O-GlcNAc transferase subunit p110  CCR4-NOT transcription complex subunit 1  Solute carrier organic anion transporter family member 4A1 | 0.00275  0.00215  0.00125  0.00035  0.00245  0.0024  5.00E-05  5.00E-05  0.00175  0.0015  0.00245  0.00045  0.0029  0.0002  0.00135  0.0005  5.00E-05  5.00E-05  0.0011  0.0014  0.0006  0.00235  5.00E-05  5.00E-05  0.0003  0.00035  0.0002  5.00E-05  0.0006  5.00E-05  0.00045  5.00E-05  0.0029  0.0008  5.00E-05  0.00045  0.0009  0.0021  0.00275  0.00105  5.00E-05  0.0001  0.0001  5.00E-05  5.00E-05  5.00E-05  5.00E-05  5.00E-05  0.00155  0.0023  0.0013  0.0003  5.00E-05  0.0001  0.0002  0.0026  5.00E-05  5.00E-05  0.0005  0.00075  0.00205  0.00015  5.00E-05  5.00E-05  0.0001  5.00E-05  0.0015  0.0013  0.002  5.00E-05  0.00015  0.00095  5.00E-05  5.00E-05  5.00E-05  5.00E-05  0.00015  5.00E-05  0.0009  5.00E-05  5.00E-05  5.00E-05  0.00255  0.0006  0.00255  5.00E-05  5.00E-05  5.00E-05  0.0001  5.00E-05  0.0015  5.00E-05  5.00E-05  5.00E-05  0.00015  0.00045  0.00105  5.00E-05  5.00E-05  5.00E-05  5.00E-05  0.0001  0.00015  5.00E-05  5.00E-05 | 0.0475234  0.0397975  0.0264593  0.0100545  0.0438818  0.0433371  0.00185882  0.00185882  0.0336609  0.0297578  0.0438818  0.012139  0.0497271  0.00610207  0.0277786  0.0133253  0.00185882  0.00185882  0.0238549  0.028411  0.0152552  0.0426082  0.00185882  0.00185882  0.008848  0.0100545  0.00610207  0.00185882  0.0152552  0.00185882  0.012139  0.00185882  0.0497271  0.0192348  0.00185882  0.012139  0.0207375  0.0392  0.0475234  0.022996  0.00185882  0.00342946  0.00342946  0.00185882  0.00185882  0.00185882  0.00185882  0.00185882  0.0306125  0.0418733  0.0268748  0.008848  0.00185882  0.00342946  0.00610207  0.0460096  0.00185882  0.00185882  0.0133253  0.0182308  0.0384288  0.0048087  0.00185882  0.00185882  0.00342946  0.00185882  0.0297578  0.0268748  0.0376511  0.00185882  0.0048087  0.0215528  0.00185882  0.00185882  0.00185882  0.00185882  0.0048087  0.00185882  0.0207375  0.00185882  0.00185882  0.00185882  0.045306  0.0152552  0.045306  0.00185882  0.00185882  0.00185882  0.00342946  0.00185882  0.0297578  0.00185882  0.00185882  0.00185882  0.0048087  0.012139  0.022996  0.00185882  0.00185882  0.00185882  0.00185882  0.00342946  0.0048087  0.00185882  0.00185882 | -0.171406  -0.193797  -0.204839  -0.216295  -0.219763  -0.224572  -0.235726  -0.235793  -0.236542  -0.246268  -0.248205  -0.248369  -0.252279  -0.258505  -0.261119  -0.263231  -0.272503  -0.277346  -0.288355  -0.290831  -0.291027  -0.291804  -0.295721  -0.295789  -0.296412  -0.29815  -0.300406  -0.300651  -0.300943  -0.302538  -0.305877  -0.305982  -0.306446  -0.310563  -0.313867  -0.31972  -0.327711  -0.330774  -0.344141  -0.344712  -0.353675  -0.354869  -0.355202  -0.36267  -0.362991  -0.36993  -0.377291  -0.38492  -0.392423  -0.395046  -0.39766  -0.39815  -0.399252  -0.409412  -0.410424  -0.41632  -0.417781  -0.42464  -0.425717  -0.445218  -0.451675  -0.459641  -0.460668  -0.464959  -0.480962  -0.484274  -0.493322  -0.509586  -0.512749  -0.514802  -0.515912  -0.524722  -0.530341  -0.532965  -0.534179  -0.535589  -0.539613  -0.558008  -0.567375  -0.574635  -0.601169  -0.615645  -0.632023  -0.643348  -0.644347  -0.645724  -0.645794  -0.662192  -0.69691  -0.704259  -0.727351  -0.738154  -0.753101  -0.757273  -0.87792  -0.881542  -0.891147  -0.89249  -0.944793  -1.12637  -1.33603  -1.62434  -1.78432  -2.10201  -2.16342 |

| 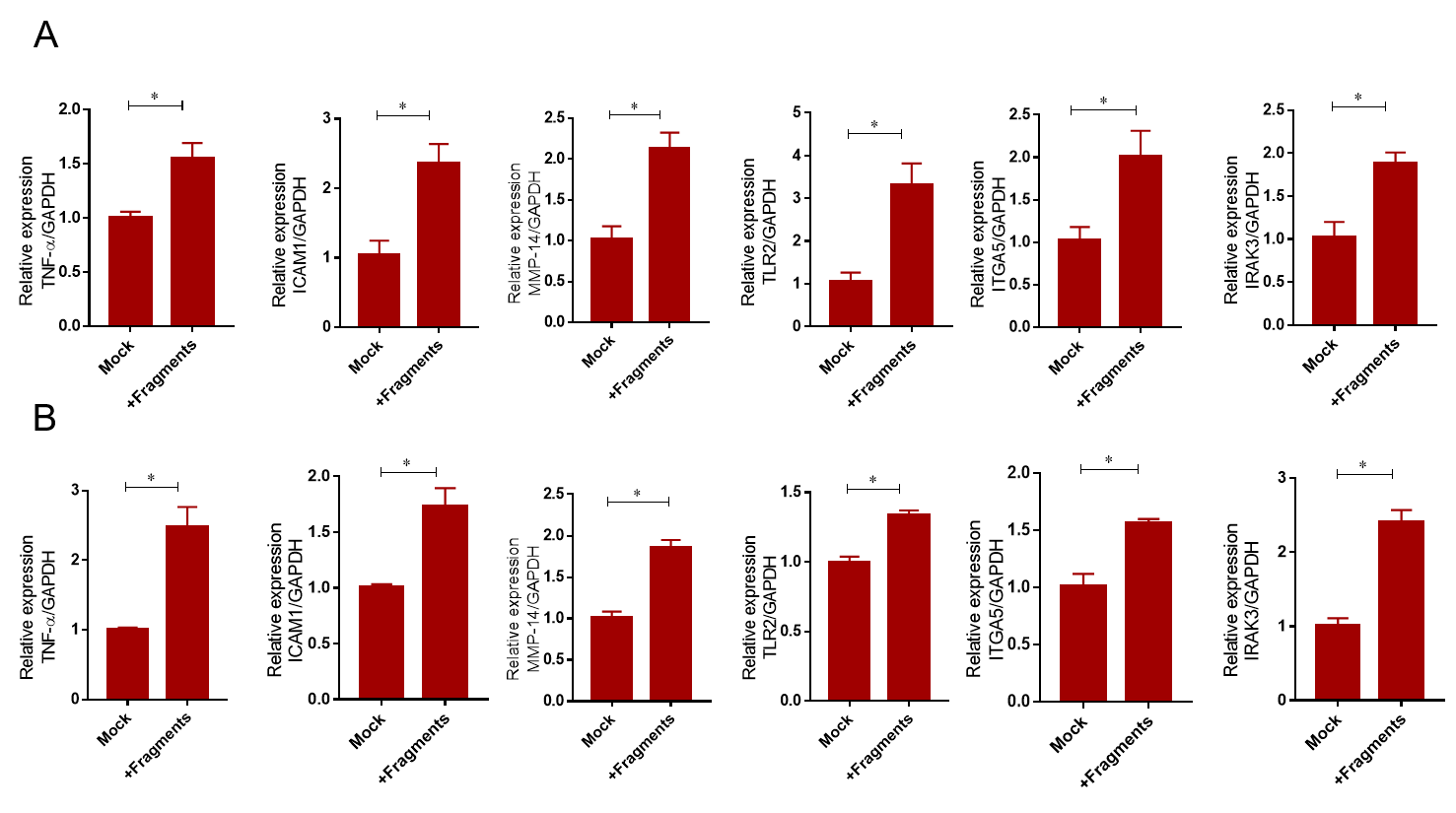 | **Figure S2.** Gene expression analysis by qRT-PCR. Murine bone marrow derived macrophages (A) and peritoneal macrophages (B) stimulated with cartilage fragments for 24 h. Target genes were selected based on RNA-seq data and assayed by qRT-PCR. The values are shown as the mean ± standard error of the mean (mean ± SEM) for *n* = 3. Statistical significances were determined using Student *t* test and were statistically significant when *P* < 0.05. *Indicates significant difference. |
| --- | --- |

**Table S3.** Gene enrichment analysis for cellular component, molecular function and biological process of upregulated genes of macrophages in response to cartilage fragments stimulation.

| **GO Term** | **GO-Term ID** | **P value** | **Gene count** | **Genes** |
| --- | --- | --- | --- | --- |
| **Cellular component (CC)** | |  |  |  |
| Extracellular exosome | GO:0070062 | 4.529E-18 | 63 | Ldha, Marcksl1, Mmp9, Fam20c, Esd, Prdx5, Rhoq, Hp, Sdc4, Prdx1, Actg1, Cd44, Tgfbi, Rnf149, Atp5h, Rhog, Pilra, Ptprj, Icam1, Pfkl, Bst2, Bst1, Myadm, Flna, Prkcb, C1qa, C1qb, Sdc1, Cd81, Cstb, Gaa, Slpi, Ctsc, Gnas, Cpd, Lilr4b, Rab10, Mvp, Fgr, C3, Aldoc, Acp2, Txn1, Mapkapk2, Tagln2, Gpi1, Tpm4, Lgals3bp, Tpi1, Rac2, Tgm2, Ehd1, Gapdh, Glrx, Ptpn6, Sppl2a, Acpp, Anxa3, Sod2, Ppia, Cyfip1, Pgk1, Cd14 |
| Focal adhesion | GO:0005925 | 8.615E-11 | 20 | Icam1, Cav1, Hck, Dock7, Mmp14, Sdc4, Tpm4, Flna, Actg1, Sdc1, Rac2, Cd44, Itga5, Ppia, Cd81, Tgm2, Adam17, Cyfip1, Rab10, Rhog |
| Extracellular matrix | GO:0031012 | 6.693E-5 | 11 | Actg1, Cfp, Lgals3bp, Mmp9, Tgfbi, Tgm2, Slpi, Mmp14, Prdx1, Gapdh, Flna |
| Cell-Cell junction | GO:0005911 | 1.250E-4 | 9 | Ptprj, Ptpn6, Itga5, Nck1, Adam17, Rab10, Myadm, Flna, Lcp2 |
| Extracellular space | GO:0005615 | 6.482E-4 | 24 | Icam1, Tnf, C3, Mmp9, Fam20c, Ccl9, Saa3, Prdx5, Hp, Pf4, Prdx1, Gpi1, Acpp, Actg1, Tpi1, Lgals3bp, Ppia, Cxcl16, Tgfbi, Cstb, Slpi, Ctsc, Cpd, Cd14 |
| **Molecular function (MF)** | | |  |  |
| Scavenger receptor activity | GO:0005044 | 0.005833 | 4 | Marco, Lgals3bp, Cxcl16, Scarf1 |
| Protein complex binding | GO:0032403 | 0.006187 | 9 | Icam1, Cdkn1a, Hif1a, Mmp9, Hcls1, Nfkbia, Cyfip1, Cpd, Flna |
| Integrin binding | GO:0005178 | 0.007203 | 5 | Icam1, Itga5, Tgfbi, Adam17, Mmp14 |
| Protein kinase C binding | GO:0005080 | 0.008945 | 4 | Plek, Sdc4, Flna, Prkcb |
| Kinase | GO:0016301 | 0.009274 | 11 | Ikbke, Irak3, Pfkl, Fgr, Nck1, Hck, Fam20c, Nadk, Mapkapk2, Prkcb |
| **Biological process (BP)** | | | | |
| Cell activation | GO:0001775 | 1.770E-27 | 58 | Cybb,Cdkn1a,Lst1,Gpi1,Flna,Tarm1,Anxa3,Ctsc,Fpr1,Fpr2,Icam1,Acpp,Gmfg,Actg1,Pf4,Cfp,Nck1,Cav1,Bcl3,Pfkl,Lcp2,Ppia,Nlrp3,Ifnar1,Rhog,Rab10,Clec4e,Ptpn6,Cyfip1,Cd14,Ptprj,Hp,Bst1,Bst2,Tlr2,Clptm1,Prdx1,Slpi,Cd44,Cstb,Aif1,C1qa,Prkcb,C3,Cd81,Tnf,Adam17,Gnas,Fgr,Plek,Mmp9,Mmp14,Aldoc,Il2rg,Sdc4,Gaa,Rac2 |
| Leukocyte activation | GO:0045321 | 2.007E-25 | 53 | Cybb,Cdkn1a,Lst1,Gpi1,Tarm1,Anxa3,Ctsc,Fpr1,Fpr2,Icam1,Acpp,Gmfg,Cfp,Nck1,Cav1,Bcl3,Pfkl,Lcp2,Ppia,Nlrp3,Ifnar1,Rhog,Rab10,Clec4e,Ptpn6,Cyfip1,Cd14,Ptprj,Hp,Bst1,Bst2,Tlr2,Clptm1,Prdx1,Slpi,Cd44,Cstb,Aif1,C1qa,Prkcb,C3,Cd81,Tnf,Adam17,Fgr,Mmp9,Mmp14,Aldoc,Il2rg,Mvp,Sdc4,Gaa,Rac2 |
| Secretion | GO:0046903 | 4.589E-24 | 58 | Cybb,Gpi1,Flna,Tarm1,Hif1a,Gapdh,Anxa3,Ctsc,Fpr1,Fpr2,Acpp,Gmfg,Pf4,Cfp,Cav1,Pfkl,Lcp2,Ppia,Nlrp3,Lgals3bp,Ifnar1,Rhog,Rab10,Clec4e,Ptpn6,Cyfip1,Cd14,Ptprj,Hp,Bst1,Bst2,Glrx,Tlr2,Slpi,Cd44,Nadk,Cstb,Aif1,Kcnn4,Tmsb4x,Prkcb,C3,Tnf,Tagln2,Tnfaip2,Gnas,Fgr,Plek,Mmp9,Aldoc,Mvp,M6pr,Sdc1,Hck,Sdc4,Gaa,Rac2,Slc16a10 |
| Defense response | GO:0006952 | 7.521E-24 | 57 | Cybb,Flna,Tarm1,Hif1a,Gapdh,Mapkapk2,Anxa3,Ctsc,Fpr1,Fpr2,Icam1,Aoah,Tnip3,Socs3,Ptges,Actg1,Birc3,Pf4,Cfp,Cxcl16,Cav1,Bcl3,Ptgir,Nlrp3,Lgals3bp,Ifnar1,Tgm2,Clec4e,Ptpn6,Cd14,Hp,Ikbke,Bst1,Bst2,Nfkbia,Tlr2,Prdx1,Slpi,Cd44,Aif1,Kcnn4,C1qa,C1qb,Tmsb4x,Irak3,C3,Cd81,Tnf,Adam17,Cmklr1,Fgr,Mmp9,Prdx5,Marco,Sdc1,Hck,Neat1 |
| Exocytosis | GO:0006887 | 2.618E-23 | 44 | Cybb,Gpi1,Flna,Tarm1,Anxa3,Ctsc,Fpr1,Fpr2,Acpp,Gmfg,Pf4,Cfp,Pfkl,Ppia,Lgals3bp,Rhog,Rab10,Ptpn6,Cyfip1,Cd14,Ptprj,Hp,Bst1,Bst2,Glrx,Tlr2,Slpi,Cd44,Cstb,Tmsb4x,Prkcb,C3,Tagln2,Tnfaip2,Fgr,Plek,Mmp9,Aldoc,Mvp,Sdc1,Hck,Sdc4,Gaa,Rac2 |
| Immune effector | GO:0002252 | 3.551E-23 | 49 | Cybb,Gpi1,Flna,Tarm1,Anxa3,Ctsc,Fpr1,Fpr2,Icam1,Acpp,Gmfg,Actg1,Birc3,Cfp,Nck1,Bcl3,Pfkl,Ppia,Nlrp3,Ifnar1,Rhog,Rab10,Clec4e,Ptpn6,Cyfip1,Cd14,Ptprj,Hp,Bst1,Bst2,Tlr2,Prdx1,Slpi,Cd44,Cstb,C1qa,C1qb,Irak3,C3,Cd81,Tnf,Adam17,Fgr,Mmp9,Aldoc,Mvp,Hck,Gaa,Rac2 |
| Membrane fusion | GO:0061025 | 7.716E-23 | 46 | Cybb,Gpi1,Flna,Tarm1,Anxa3,Ctsc,Fpr1,Fpr2,Acpp,Gmfg,Pf4,Cfp,Pfkl,Ppia,Lgals3bp,Rhog,Rab10,Ptpn6,Cyfip1,Cd14,Ptprj,Hp,Bst1,Bst2,Glrx,Tlr2,Ehd1,Slpi,Cd44,Cstb,Tmsb4x,Prkcb,C3,Cd81,Tagln2,Tnfaip2,Fgr,Plek,Mmp9,Aldoc,Mvp,Sdc1,Hck,Sdc4,Gaa,Rac2 |
| Vesicle fusion | GO:0006906 | 1.290E-22 | 44 | Cybb,Gpi1,Flna,Tarm1,Anxa3,Ctsc,Fpr1,Fpr2,Acpp,Gmfg,Pf4,Cfp,Pfkl,Ppia,Lgals3bp,Rhog,Rab10,Ptpn6,Cyfip1,Cd14,Ptprj,Hp,Bst1,Bst2,Glrx,Tlr2,Slpi,Cd44,Cstb,Tmsb4x,Prkcb,C3,Tagln2,Tnfaip2,Fgr,Plek,Mmp9,Aldoc,Mvp,Sdc1,Hck,Sdc4,Gaa,Rac2 |
| Regulation of immune system | GO:0002682 | 4.761E-22 | 55 | Cdkn1a,Lst1,Gpi1,Tarm1,Hif1a,Pilra,Mapkapk2,Ctsc,Fpr1,Fpr2,Icam1,Tnip3,Socs3,Actg1,Birc3,Pf4, Cfp,Nck1,Cav1,Lcp2,Nlrp3,Clec4e,Ptpn6,Cyfip1,Sppl2a,Cd14,Ptprj,Ikbke,Bst1,Bst2,Nfkbia,Tlr2,Clptm1,Cd44,Aif1,Kcnn4,C1qa,C1qb,Prkcb,Irak3,C3,Cd81,Tnf,Adam17,Cmklr1,Gnas,Fgr,Mmp9,Mmp14,Il2rg,Marco,Hck,Sdc4,Hcls1, Rac2 |
| Inflammatory response | GO:0006954 | 4.438E-16 | 35 | Cybb,Hif1a,Mapkapk2,Ctsc,Fpr1,Fpr2,Icam1,Aoah,Tnip3,Socs3,Ptges,Birc3,Pf4,Ptgir,Nlrp3,Tgm2,Cd14,Hp,Bst1,Nfkbia,Tlr2,Cd44,Aif1,C1qa,Tmsb4x,C3,Cd81,Tnf,Adam17,Cmklr1,Mmp9,Prdx5,Sdc1,Hck,Neat1 |

**Table S4.** Gene enrichment analysis for mouse response phenotypes of upregulated genes of macrophages in response to cartilage fragments stimulation.

| **GO Term** | **GO-Term ID** | **P value** | **Gene count** | **Genes** |
| --- | --- | --- | --- | --- |
| Abnormal innate immunity | MP:0002419 | 1.361E-24 | 43 | Cybb,Cdkn1a,Hif1a,Pilra,Mapkapk2,Ctsc,Fpr1,Fpr2,Icam1,Socs3,Ptges,Cfp,Marcksl1,Cav1,Bcl3,Lcp2,Nlrp3,Ifnar1,Scarf1,Tgm2,Clec4e,Ptpn6,Cd14,Ptprj,Hp,Nfkbia,Tlr2,Prdx1,Slpi,Cd44,Irak3,C3,Cd81,Tnf,Adam17,Cmklr1,Fgr,Mmp9,Adgre1,Il2rg,Marco,Hck,Rac2 |
| Abnormal macrophage physiology | MP:0002451 | 4.102E-21 | 34 | Cybb,Cdkn1a,Hif1a,Mapkapk2,Fpr2,Icam1,Socs3,Ptges,Marcksl1,Cav1,Bcl3,Nlrp3,Ifnar1,Scarf1,Tgm2,Clec4e,Ptpn6,Cd14,Ptprj,Hp,Nfkbia,Tlr2,Slpi,Cd44,Irak3,C3,Cd81,Tnf,Adam17,Cmklr1,Fgr,Adgre1,Marco,Hck |
| Abnormal professional antigen presenting cell physiology | MP:0002452 | 5.869E-20 | 47 | Cybb,Cdkn1a,Hif1a,Mapkapk2,Fpr2,Icam1,Socs3,Ptges,Marcksl1,Cav1,Bcl3,Lcp2,Ppia,Nlrp3,Ifnar1,Scarf1,Rhog,Tgm2,Clec4e,Ptpn6,Sppl2a,Csf2rb,Cd14,Ptprj,Hp,Bst1,Nfkbia,Tlr2,Slpi,Cd44,Prkcb,Irak3,C3,Cd81,Tnf,Adam17,Cmklr1,Fgr,Mmp9,Adgre1,Mmp14,Il2rg,Marco,Sdc1,Hck,Hcls1,Rac2 |

**Table S5.** Gene enrichment analysis for human diseases terms of upregulated genes of macrophages in response to cartilage fragments stimulation.

| **GO Term** | **GO-Term ID** | **P value** | **Gene count** | **Genes** |
| --- | --- | --- | --- | --- |
| Rheumatoid Arthritis | C0003873 | 2.458E-14 | 46 | Cybb,Cdkn1a,Gpi1,Hif1a,Gapdh,Anxa3,Fpr2,Icam1,Socs3,Ptges,Pf4,Cxcl16,Bcl3,Lcp2,Ppia,Nlrp3,Lgals3bp,Txn1,Tgfbi,Tgm2,Ptpn6,Cd14,Ptprj,Hp,Ikbke,Bst1,Nfkbia,Tlr2,Cd44,Aif1,C3,Tnf,Adam17,Tnfaip2,Cmklr1,Plek,Mmp9,Prdx5,Mmp14,Il2rg,Marco,Sdc1,Hcls1,Sod2,Rac2,Tpi1 |
| Degenerative polyarthritis | C0029408 | 5.431E-14 | 34 | Cdkn1a,Gpi1,Hif1a,Gapdh,Esd,Icam1,Socs3,Ptges,Cxcl16,Cav1,Nlrp3,Tgfbi,Tgm2,Fam20c,Ptpn7,Cd14,Hp,Glrx,Tlr2,Slpi,Cd44,C3,Tnf,Adam17,Cmklr1,Mmp9,Prdx5,Mmp14,Il2rg,Mvp,Sdc1,Sdc4,Sod2,Tpi1 |
| Amyloidosis | C0002726 | 7.701E-10 | 31 | Cybb,Gapdh,Fpr1,Fpr2,Icam1,Socs3,Cav1,Ldha,Ppia,Nlrp3,Txn1,Tgfbi,Cd14,Hp,Tlr2,Cd44,Cstb,Aif1,C3,Tnf,Adam17,Cmklr1,Mmp9,Prdx5,Mmp14,Mvp,Sdc1,Hcls1,Gaa,Sod2,Tpi1 |
| Inflammatory bowel Disease | C0021390 | 1.156E-12 | 28 | Cybb,Lst1,Hif1a,Gapdh,Pilra,Fpr2,Icam1,Socs3,Cxcl16,Bcl3,Nlrp3,Ifnar1,Acsl1,Fam20c,Cd14,Hp,Nfkbia,Tlr2,Cd44,Kcnn4,C3,Tnf,Adam17,Mmp9,Mmp14,Sdc1,Rac2,Tpi1 |
| Multiple Myeloma | C0026764 | 1.86E-11 | 38 | Cdkn1a,Hif1a,Cd52,Gapdh,Slc31a2,Mapkapk2,Icam1,Socs3,Birc3,Pf4,Marcksl1,Cav1,Bcl3,Itga5,Ldha,Ppia,Slc25a37,Ifnar1,Txn1,Ptpn6,Cd14,Ptprj,Bst2,Slc2a6,Nfkbia,Tlr2,Cd44,Tmsb4x,Cd81,Tnf,Adam17,Mmp9,Il2rg,Mvp,Sdc1,Hck,Sod2,Neat1 |
| Sepsis | C0243026 | 3.140E-11 | 23 | Cybb,Hif1a,Gapdh,Fpr2,Icam1,Aoah,Tnip3,Socs3,Cav1,Nlrp3,Tgfbi,Cd14,Hp,Tlr2,Cd44,Tmsb4x,Irak3,Tnf,Adam17,Mmp9,Marco,Sdc1,Sod2 |

**Table S6.** Gene enrichment for KEGG pathways of regulated genes of macrophages in response to cartilage fragments stimulation.

|  | |  | |  | |  | |  |
| --- | --- | --- | --- | --- | --- | --- | --- | --- |
| **GO Term** | **GO-Term ID** | | **P value** | | **Gene count** | | **Genes** | |
| Proteoglycans in cancer | mmu05205 | | 1.203E-7 | | 15 | | Ptpn6, Cav1, Tnf, Hcls1, Mmp9, Tlr2, Sdc4, Flna, Prkcb, Actg1, Cdkn1a, Sdc1, Hif1a, Cd44, Itga5 | |
| Gluconeogenesis | mmu00010 | | 1.347E-4 | | 7 | | Tpi1, Ldha, Pfkl, Aldoc, Pgk1, Gpi1, Gapdh | |
| TNF signaling pathway | mmu04668 | | 3.293E-4 | | 8 | | Icam1, Tnf, Socs3, Mmp9, Bcl3, Nfkbia, Birc3, Mmp14 | |
| Toll-like receptor signaling pathway | mmu04620 | | 0.002714 | | 6 | | Ikbke, Tnf, Tlr2, Nfkbia, Cd14, Ifnar1 | |
| Phagosome | mmu04145 | | 0.007382 | | 7 | | Actg1, Marco, C3, Itga5, Tlr2, M6pr, Cd14 | |
| Carbon metabolism | mmu01200 | | 0.009630 | | 7 | | Tpi1, Pfkl, Aldoc, Esd, Pgk1, Gpi1, Gapdh | |

**Table S7.** Primers used in this study.

| **Target** | **Forward** | **Reverse** |
| --- | --- | --- |
| ***GAPDH*** | AGGTCGGTGTGAACGGATTTG | GGGGTCGTTGATGGCAACA |
| ***IRAK3*** | GATCCTGGACAGCTGCGAT | CTGTTTGAAAGTCGCTCCGC |
| ***MMP-14*** | ATGGCCCCCTTTTACCAGTG | TTGTGGGTGACCCTGACTTG |
| ***ICAM1*** | GTGGGTCGAAGGTGGTTCTT | CAGTGTGAATTGGACCTGCG |
| ***MARCO*** | ACTCCAGAGGGAGAGCACTT | TCTCTGTGCCCCGACAATTC |
| ***PTGES*** | CGCAACGACATGGAGACAATC | TATCCAGGCGATCAGAGGGT |
| ***TLR2*** | TGCTACGAGCTCTTTGGCTC | GCAGAACAGCGTTTGCTGAA |
| ***ITGA5*** | ATGAAGGCAGGCACCAGTC | CAGGTTCTTGCTGAGGATCTGA |
| ***TNF-α*** | AGCCGATGGGTTGTACCTTG | ATAGCAAATCGGCTGACGGT |
